# Mapping the Neural Basis of Neuroeconomics with Functional Magnetic Resonance Imaging: A Narrative Literature Review

**DOI:** 10.3390/brainsci14050511

**Published:** 2024-05-18

**Authors:** Carlo A. Mallio, Andrea Buoso, Massimo Stiffi, Laura Cea, Daniele Vertulli, Caterina Bernetti, Gianfranco Di Gennaro, Martijn P. van den Heuvel, Bruno Beomonte Zobel

**Affiliations:** 1 Fondazione Policlinico Universitario Campus Bio-Medico, 00100 Rome, Italy; andrea.buoso@unicampus.it (A.B.); massimo.stiffi@unicampus.it (M.S.); laura.cea@unicampus.it (L.C.); daniele.vertulli@unicampus.it (D.V.); c.bernetti@policlinicocampus.it (C.B.); b.zobel@policlinicocampus.it (B.B.Z.); 2 Research Unit of Radiology, Department of Medicine and Surgery, Università Campus Bio-Medico di Roma, 00100 Rome, Italy; 3 Department of Health Sciences, Medical Statistics, University of Catanzaro “Magna Græcia”, 88100 Catanzaro, Italy; gianfranco.digennaro@unicz.it; 4 Department of Complex Trait Genetics, Center for Neurogenomics and Cognitive Research, Vrije Universiteit Amsterdam, Amsterdam Neuroscience, 1081 Amsterdam, The Netherlands; martijn.vanden.heuvel@vu.nl; 5 Department of Child and Adolescent Psychiatry and Psychology, Amsterdam UMC, Vrije Universiteit Amsterdam, 1081 Amsterdam, The Netherlands

**Keywords:** fMRI, brain, neuroeconomics, subjective value

## Abstract

Neuroeconomics merges neuroscience, economics, and psychology to investigate the neural basis of decision making. Decision making involves assessing outcomes with subjective value, shaped by emotions and experiences, which are crucial in economic decisions. Functional MRI (fMRI) reveals key areas of the brain, including the ventro-medial prefrontal cortex, that are involved in subjective value representation. Collaborative interdisciplinary efforts are essential for advancing the field of neuroeconomics, with implications for clinical interventions and policy design. This review explores subjective value in neuroeconomics, highlighting brain regions identified through fMRI studies.

## 1. Introduction

Neuroeconomics, an intriguing field at the intersection of neuroscience, economics, and psychology, is related to how real-time neural activity underpins various decision-making processes [1]. From evaluating options and assessing risks and rewards to making choices and interacting strategically with others, neuroeconomics sheds light on the intricate neural mechanisms governing these behaviors [1]. This interdisciplinary approach has significantly advanced our understanding of decision making across a spectrum of situations, including those involving uncertainty (e.g., choosing between a risky gamble and a guaranteed smaller reward) [2], intertemporal choice (e.g., prioritizing immediate gratification over long-term benefits) [2], and game theory (e.g., anticipating and strategically responding to the actions of others) [2].

Founded in 1999, neuroeconomics laid the groundwork for investigating the biological underpinnings of decision making in economic contexts [3]. Pioneering studies using animals, such as monkeys, demonstrated that decisions are made by evaluating the relative value assigned to each potential response [3]. Notably, these studies identified specific brain regions exhibiting neural activity directly linked to the probability of a particular response leading to a reward [3]. Building upon these findings, researchers extended the inquiry to humans by employing neuroimaging techniques within the framework of neuroeconomics and decision-making research [4,5,6,7].

A core objective of neuroeconomics research is to establish a biological model of decision making in economic environments. This ambitious goal is pursued by combining sophisticated experimental designs, advanced data analysis methods, and powerful functional brain imaging techniques, with functional magnetic resonance imaging (fMRI) being a prominent tool [8].

Decision making in neuroeconomics extends beyond a purely rational, calculated approach. This field recognizes the significant influence of emotional, social, and neurological factors on how people make choices within economic contexts [9]. A cornerstone concept in neuroeconomic decision making is subjective value, which refers to the value an individual assigns to a good, service, or experience based on their unique preferences, wants, and needs [10]. This concept emphasizes that value is not an objective property inherent to an object itself, but rather, a dynamic construct shaped by the individual’s perception and evaluation during the decision-making process [10].

Non-invasive neuroimaging techniques such as fMRI play a crucial role in enabling researchers to observe and analyze the activity of brain regions and their interactions during decision-making tasks [2,11,12]. Brain fMRI studies typically involve either examining brain activity at rest (resting-state fMRI) or comparing brain images of participants engaged in different tasks, both experimental and control conditions [2,11,12]. By analyzing these differential brain activation patterns under specific conditions, researchers gain valuable insights into the neural correlates of decision making [2,11,12].

The applications of fMRI extend beyond the domain of neuroeconomics. It has established itself as a valuable tool in various medical fields. For instance, fMRI is utilized in pre-surgical planning to map critical brain areas responsible for functions like speech and movement, thereby minimizing the risk of surgical damage [13,14]. Additionally, fMRI can be of value in neurological conditions like Alzheimer’s disease, and it has even been used in attempts to assess the effectiveness of novel medications for psychiatric disorders [15,16].

The potential of fMRI extends further, with ongoing investigations into how our brains make decisions possibly contributing to potential future advancements in decision-making improvement strategies across various domains [4,5,6,7]. Fueled by this potential, neuroscientists are actively using fMRI to explore the intricate interplay between brain function, decision making, and subjective value in humans.

The aim of the present paper is to provide an overview of the subjective value in neuroeconomics, highlighting the key brain regions identified through fMRI studies.

## 2. Decision Making, Subjective Value, and fMRI

Decision making, the selection of actions based on the likelihood and potential value of outcomes, necessitates the synchronized interplay of motivational, emotional, and cognitive networks within the brain. These networks integrate information to formulate and evaluate potential choices [17,18].

Central to all forms of decision making is the cost–benefit analysis occurring within specific brain regions. This analysis is crucial because optimal decisions, regardless of the number of options, must be made under conditions of limited information [17,18]. This process involves a complex relationship within the prefrontal cortex, a region associated with higher-order cognitive functions [19,20,21,22]. Specifically, the dorso-lateral prefrontal cortex (DLPFC) is critical for evaluating options and their potential consequences, while the orbito-frontal cortex (OFC) assigns value to those options based on past experiences and emotional influences [19,20,21,22]. The anterior cingulate cortex (ACC) plays a role in signaling decision conflict, and the posterior cingulate cortex (PCC) is involved in integrating information from various brain regions to guide the final choice [19,20,21,22].

By investigating these neural underpinnings of economic decision making, neuroeconomics offers a deeper understanding of how emotions, cognition, and the workings of specific brain regions interact to shape our choices, even in the face of uncertainty [19,20,21,22]. These findings have the potential not only to refine economic models but also to inform various fields, from individual financial behavior to the design of effective incentive structures.

Subjective value is fundamental in several fields, such as economics, psychology, neuroscience, and neuroeconomics [23].

In economics, the theory of subjective value is based on the concept that the value of a good service is determined by people’s subjective perception and evaluation rather than by objective measurements such as the labor employed in its production. Understanding subjective value is crucial for explaining complex economic behaviors, such as investment choices, purchasing behaviors, and consumption preferences [23].

In psychology, subjective value is related to cognitive psychology and psychology theory.

The concept of subjective value in neuroeconomics, demonstrated through studies investigating brain activities during decision making, refers to an individual’s perception of the value of a given stimulation or experience [24,25,26].

This perspective considers the fact that economic and financial decisions are not always rational and that emotional and cognitive factors play a significant role in decision making [24,25,26]. For example, an object or opportunity may be evaluated differently by different people due to variations in neural responses related to emotions, expectations, and past experiences.

Furthermore, subjective value is influenced by risk and ambiguity related to the decision-making process [27,28,29,30]. While economics traditionally focused on objective factors like production cost, in subjective value, the concept of a common scale becomes crucial. It is a personal decision-making framework, a way to weigh all these subjective factors against each other to find the option that delivers the greatest overall subjective benefit. In essence, value maximization, when considering subjective value, is about finding the option that brings the greatest satisfaction and reward, considering the unique lens through which you perceive value [31,32,33].

Under these circumstances subjective value is simply proportional to the expected utility of each choice. Hence, subjective value is the overall value of a certain option for an individual subject, considering any possible parameter, including risks, probability, and ambiguity.

Subjective value in neuroeconomics emphasizes the importance of considering the individual and psychological dimensions in economic decisions, helping to develop a more complete and realistic view of human decision making [31,32,33].

fMRI is a widely used neuroimaging technique to measure brain activation [11,12]. Its non-invasiveness, absence of radiation exposure, and relatively wide availability makes it an ideal method for examining inter-individual differences and variation in brain activity and dynamics, including the measurement of subjective value in the field of neuroeconomics.

fMRI is a technique sensitive to changes in oxygen levels in the blood, with fluctuations coupled with the level of neural activation in brain areas during the resting state (i.e., when the brain is at rest [11,12]) or in response to performing specific functions (examples: sensory processing, working-memory, language, decision making) [34,35,36]. The fMRI signal, defined as blood oxygen level-dependent (BOLD) contrast, is an indirect measure of brain activity. This means the fMRI signal does not directly measure the activity of neurons, but rather, reflects changes in blood flow and oxygen levels that occur when brain regions become more active [34,35,36].

This technique allows researchers to obtain both temporal and spatially high-resolution images of brain activation, providing a deeper understanding of the neural basis of behavior. Functional MRI can perform simultaneous, though indirect, measurements of neural activity in both the cortex as well as in deeper sub-cortical nuclei [34,35,36]. This allows us to map the regional brain activity associated with specific stimuli or tasks across almost the entire brain, providing a powerful methodology to study how brain activity is related to different behaviors in decision making.

As in the wider field of neuroscience, fMRI is a valuable tool in neuroeconomics, helping to elucidate the biological mechanisms of choice under uncertainty, across time, and in social contexts. This safe, non-invasive method allows researchers to map brain regions involved in weighing up options, experiencing emotions, and ultimately making choices [34,35,36,37,38,39,40]. It provides a powerful tool for understanding how our brains make decisions and paves the way for future advancements in decision-making improvement and related fields [34,35,36,37,38,39,40]. Indeed, fMRI can play a key role in bridging the gap between subjective experience and decision making in neuroeconomics. By measuring brain activity indirectly through blood oxygen levels, fMRI allows researchers to pinpoint which regions are activated during economic decisions [34,35,36,37,38,39,40,41,42,43,44,45,46,47,48,49,50,51,52]. This offers valuable insights into the neural basis of subjective value, helping us understand how our personal experiences, emotions, and expectations influence the choices we make. fMRI’s ability to map brain activity across different areas provides a powerful tool for deciphering the complex interplay between psychological and biological factors that shape our economic behavior.

## 3. Methods

The literature search was performed on September 2023 using MEDLINE PubMed Central, considering only articles written in English and without limits in time span.

The combination of keywords for the article search was “neuroeconomics”, “decision making”, “subjective value”, and “fMRI”. Relevant articles related to this topic were also selected from the reference list of each identified article.

## 4. Results of the Individual Studies

Major research on the topic of fMRI, decision making, and subjective value showed interesting findings (Table 1).

Chib et al. reported behavioral data confirming that participants found all good categories rewarding and assigned similar value to them, with choices made inside the scanner reflecting these valuations [34]. Moreover, this study identified a common region in the VMPFC that actively encoded a value (i.e., willingness to pay) assigned to different categories of goods (i.e., money, trinkets, snacks), regardless of the reference point used in decision making [34].

Bartra et al., in a meta-analysis, introduced the concept that distinct brain networks are involved in processing subjective value during decisions [41]. Some regions might be sensitive to intensity (i.e., high or low value), while others seem to track the overall importance of an option [41].

Clithero and Rangel, in another meta-analysis on this topic, suggested that a core valuation system is active in the brain, with distinct subnetworks and a processing flow that considers the concreteness of the reward [42].

Bakkour et al. highlighted that the hippocampus plays a significant role in processing deliberations for decisions where the value of options is similar [52]. This might involve building internal evidence or value-based arguments during the decision process [52].

Lee et al., in 2021, conducted a study challenging the role of VMPFC in representing subjective value during decisions, suggesting it might prioritize task-relevant information over a previously proposed grid-like code [39].

More recently, Zyuzin et al. underscored that a core brain region, the left ventro-medial prefrontal cortex (VMPFC), tracks value across simple and complex decisions; additional areas in the prefrontal cortex, including the DLPFC, ventro-lateral prefrontal cortex (VLPFC), and cerebellum, are recruited depending on the specific complexity of the choices being made.

Taken together, the results of individual studies on this topic highlight how complex and multifaced the activity of the human brain is during decision making and the assignment of subjective value to alternatives.

## 5. Discussion

**Figure 1:**
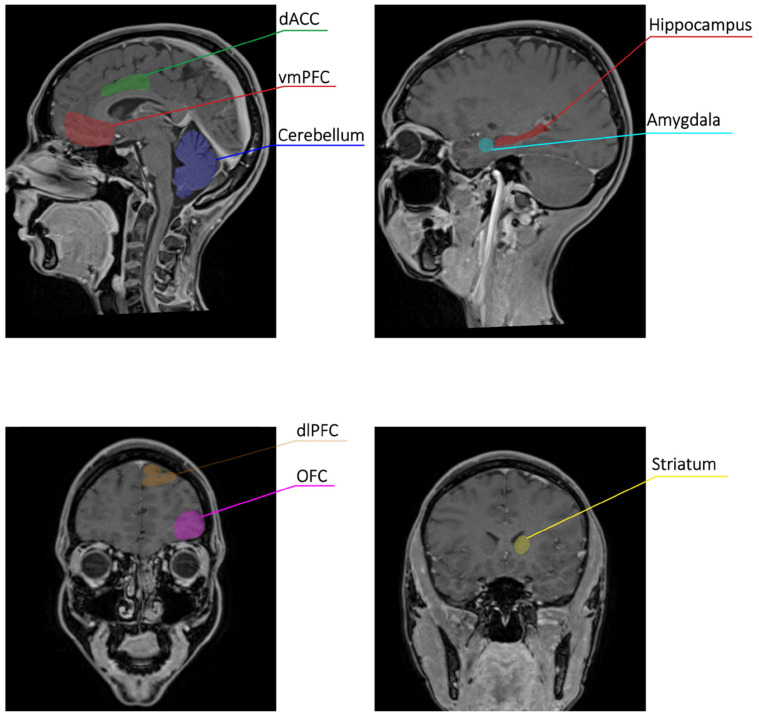
Main cerebral areas related to subjective value in decision making. This figure was built using MR T1 images, and regions of interest were placed according to the expected anatomical location of the involved areas. Abbreviations: dACC (dorsal anterior cingulate cortex), vmPFC (ventro-medial prefrontal cortex), dlPFC (dorso-lateral prefrontal cortex), OFC (orbito-frontal cortex).

Every decision we make involves a trade-off between benefits and costs. At the core of economic decision making lies the fundamental principle of maximizing subjective value, or utility—essentially, getting the most out of our choices [1]. A central question in neuroeconomics is precisely where this subjective value is represented in the brain [41,42,43,44,45,46,47,48,49,50,51,52] (Figure 1).

### 5.1. Brain Regions and Subjective Value

Functional magnetic resonance imaging (fMRI) studies reveal activation in reward-related brain regions that correlates with subjective value, regardless of its definition [34]. This suggests that the brain’s reward system plays a crucial role in influencing choices with potential positive outcomes.

fMRI studies using simple tasks identified the VMPFC, encompassing the OFC, as a key region involved in decision making based on subjective value [34,35,36,45]. Further research suggests a causal link between the values encoded in the OFC and actual economic choices [46].

### 5.2. A Network for Subjective Value

However, a more complex picture emerges from broader neuroimaging studies. The DLPFC, known for its role in executive functions, is also activated during value-based decision processes [47]. Interestingly, the DLPFC and VMPFC appear to work together to represent subjective value accurately, especially in complex decisions [47,48].

The hippocampus also plays a role. This brain region is crucial for forming memories related to decisions and integrating information that influences how we evaluate options [49,50,51,52]. Memories act as a library of past experiences, shaping how we evaluate and choose in the present [53].

### 5.3. Emotions and Subjective Value

Emotions significantly influence subjective value, particularly in early decision stages. The amygdala and ACC modulate emotional responses associated with decisions, influencing how we evaluate emotionally charged stimuli [54,55]. These areas work with the prefrontal cortex and hippocampus, assigning subjective value through interactions between neural circuits [54,55].

Furthermore, the striatum, particularly the nucleus accumbens, is involved in representing rewards and may influence subjective value by biasing choices towards pleasure and reward [56,57,58].

### 5.4. Distinct Networks for Different Tasks

These brain regions work together during subjective value-based decision making (Figure 1 and Table 1). A meta-analysis of 206 fMRI studies suggests two distinct brain activity patterns [41]. One network focuses on reward, motivation, and calculating subjective value. Areas like the VMPFC, PCC, and ventral striatum activate when considering potential rewards and their desirability.

The other network emphasizes risk assessment and the importance of an option. The DLPFC, insula, and dorsal/caudal striatum become active when weighing up the potential risks and significance of a choice.

In essence, the brain utilizes distinct networks to process different aspects of a decision, considering the rewards and associated risks to guide informed choices.

### 5.5. Limitations of fMRI

Despite the advancements in fMRI, limitations exist. Firstly, it measures indirect indicators of brain activity, such as blood flow and oxygenation changes [59,60]. While these measures correlate with neural activity, they do not directly reflect neuronal firing. Secondly, fMRI’s temporal resolution is limited, unable to capture the rapid dynamics of neural processes involved in decision making [59,60].

Another challenge is synthesizing research findings due to the significant heterogeneity across studies [34,39,41,42,47,52]. Variations in methodology, participant samples, and the diversity of fMRI tasks employed make direct comparisons difficult. Standardized approaches are needed to establish a more cohesive understanding of the neuroeconomics of decision making.

These limitations highlight the importance of using fMRI results cautiously, alongside other neuroimaging techniques and behavioral data, to gain a more comprehensive picture of brain function.

### 5.6. Future Perspectives

In the field of neuroeconomics and decision making, the future holds promising avenues for exploration, particularly in addressing the multifaceted dynamics of large-scale age differences across people’s lifespans [61]. Understanding how decision-making processes evolve and are influenced by age-related factors will be crucial, shedding light on cognitive changes and their impact on economic behaviors.

Moreover, with the prevalence of neurodegenerative diseases such as Alzheimer’s and Parkinson’s on the rise, there is a pressing need to delve deeper into how these conditions intersect with decision-making abilities [62,63]. Exploring the neural mechanisms underlying decision making in individuals affected by such diseases can provide invaluable insights into their cognitive decline and potential interventions.

The integration of advanced neuroimaging techniques like fMRI, EEG, and MEG with emerging technologies such as artificial intelligence (AI) could revolutionize this field [64,65,66,67,68]. These synergies offer unprecedented opportunities to decode the intricacies of decision-making processes with unprecedented precision and depth. AI algorithms can analyze vast amounts of data, uncovering patterns and correlations that might overcome human observation alone [64,65,66,67,68], possibly enhancing our understanding of the neural basis of economic decision making.

Furthermore, this integration might enable the development of innovative interventions and personalized treatments for individuals with neurocognitive disorders. By leveraging AI-driven predictive models based on neuroimaging data, clinicians can tailor interventions to suit the specific cognitive profiles and needs of patients, ultimately improving their quality of life.

In summary, the future of neuroeconomics and decision making is likely to rely on the junction of neuroscience, technology, and interdisciplinary collaboration. By addressing the complexities of age-related differences and neurodegenerative diseases, and leveraging cutting-edge techniques and AI-driven insights, researchers are poised to unlock new frontiers in understanding human decision making and its neural underpinnings.

## 6. Conclusions

The investigation of subjective value in neuroeconomics, facilitated by fMRI, offers valuable insights into the neural mechanisms underlying economic decisions. Integrating economic, psychological, and neuroscientific principles enhances our understanding of how the brain evaluates options in economic contexts. These insights hold promise for clinical interventions targeting disorders affecting subjective value assessment and for improving public policy through more effective incentive design. Further advancements in technology and interdisciplinary collaboration are key to advancing this field and its current and future broad applications.

## Figures and Tables

**Table 1:** Key feature of the major papers exploring the subjective value in decision making with fMRI.

Authors	Year	Article Type	Field Strength	Participants	fMRI Conditions	Statistical Approach	Brain Regions	Major Conclusions
Chib et al. [34]	2009	Original article	3T	32 healthy subjects	Decision making (3 good classes)	General linear model analysis	vmPFC	vmPFC encodes value independent of good category.
Bartra et al. [41]	2013	Meta-analysis	-	206 publications	-	Coordinate-based meta-analysis	Anterior insula, dmPFC, dorsal/posterior striatum, thalamus, vmPFC, ventral striatum	Regions form an “evaluation system” for value-based decisions.
Clithero & Rangel [42]	2014	Meta-analysis	3T	81 publications	-	Random-effects parallel image-based meta-analysis	vmPFC, ventral striatum, PCC	vmPFC, ventral striatum, and PCC are central for value computation. Subnetworks exist within vmPFC.
Bakkour et al. [52]	2019	OriginalArticle	3T	Exp 1: 30 healthy subjects; Exp 2: 6 amnesia patients and 14 controls	Food choice, color dots, memory recognition	General linear model analysis	Hippocampus	Hippocampus activity increases with deliberation time. Damage leads to more random choices.
Lee et al. [39]	2021	Original article	3T	145 (session 1), 102 (session 2)	Reward-guided decision making	General linear model analysis	vmPFC	vmPFC activity reflects subjective value, not a fixed grid-like signal.
Zyuzin et al. [47]	2023	Original article	3T	68 healthy subjects	Pairwise comparisons (varying complexity)	General linear model analysis	vmPFC, dlPFC, vlPFC, cerebellum, lPFC	vmPFC and DLPFC track value. Task-specific regions activated for complex decisions.

Abbreviations: dlPFC or DLPFC (dorso-lateral prefrontal cortex), dmPFC (dorso-medial prefrontal cortex), fMRI (functional magnetic resonance imaging), lPFC (lateral prefrontal cortex), PCC (posterior cingulate cortex), vlPFC (ventro-lateral prefrontal cortex), vmPFC or vmPFC (ventro-medial prefrontal cortex).

## Abbreviations

ACC	anterior cingulate cortex
aPFC	anterior prefrontal cortex
BOLD	blood oxygenation level-dependent
dACC	dorsal anterior cingulate cortex
dlPFC or DLPFC	dorso-lateral prefrontal cortex
dmPFC	dorso-medial prefrontal cortex
fMRI	functional magnetic resonance imaging
lPFC	lateral prefrontal cortex
MRI	magnetic resonance imaging
OFC	orbito-frontal cortex
PCC	posterior cingulate cortex
SV	subjective value
vlPFC	ventro-lateral prefrontal cortex
vmPFC or VMPFC	ventro-medial prefrontal cortex
AI	artificial intelligence
